# Renal histopathological lesions after liver transplantation: What can we find besides calcineurin inhibitor-induced nephrotoxicity?

**DOI:** 10.1186/s12882-022-02952-y

**Published:** 2022-09-30

**Authors:** Haijiao Jin, Yuehan Wei, Yongbing Qian, Jiang Zhang, Yao Xu, Hang Zhou, Minfang Zhang, Wenyan Zhou, Chaojun Qi, Wei Jin, Shan Mou, Qin Wang, Jianjun Zhang

**Affiliations:** 1 Department of Nephrology, Ren Ji Hospital, Shanghai Jiao Tong University School of Medicine, Pujian Road 160, 200127 Shanghai, China; 2 Department of Liver Surgery, Ren Ji Hospital, Shanghai Jiao Tong University School of Medicine, Pujian Road 160, 200127 Shanghai, China

**Keywords:** Liver transplantation, Renal biopsy, Chronic kidney disease, Calcineurin inhibitor toxicity

## Abstract

**Background:**

Chronic kidney disease (CKD) is a common complication after liver transplantation and is traditionally considered to be secondary to calcineurin inhibitors (CNIs). However, several studies have reported that the etiology of CKD after liver transplantation is broad and may only be assessed accurately by renal biopsy. The current study aimed to explore the usefulness of renal biopsies in managing CKD after liver transplantation in daily clinical practice.

**Method:**

This retrospective analysis enrolled all post-liver transplantation patients who had a renal biopsy in a single center from July 2018 to February 2021.

**Results:**

Fourteen renal biopsies were retrieved for review from 14 patients at a median of 35.7 (minimum-maximum: 2.80–134.73) months following liver transplantation. The male-to-female ratio was 13:1 (age range, 31–75 years). The histomorphological alterations were varied. The predominant glomerular histomorphological changes included focal segmental glomerular sclerosis (FSGS) (n = 4), diabetic glomerulopathy (n = 4), and membranoproliferative glomerulonephritis (n = 4). Thirteen (92.9%) patients had renal arteriolar sclerosis. Immune complex nephritis was present in six patients, of whom only two had abnormal serum immunological indicators. Despite interstitial fibrosis and tubular atrophy being present in all the patients, only six (42.9%) presented with severe interstitial injury. No major renal biopsy-related complications occurred. After a mean follow-up of 11.8 months (range: 1.2–29.8), three patients progressed to end-stage renal disease (ESRD).

**Conclusion:**

The etiology of CKD after liver transplantation might be more complex than originally thought and should not be diagnosed simply as calcineurin inhibitors(CNI)-related nephropathy. Renal biopsy plays a potentially important role in the diagnosis and treatment of CKD after liver transplantation and might not be fully substituted by urine or blood tests. It may help avoid unnecessary changes to the immunosuppressants and inadequate treatment of primary diseases.

## Introduction

Chronic kidney disease (CKD) is a common problem after liver transplantation. The cumulative incidence of CKD after liver transplantation is 8.0%, 13.9%, and 18.1% over 1, 3, and 5 years, respectively[1]. Studies evaluating renal pathology after liver transplantation are sparse, and sample sizes are small[2–10]. Post-liver transplantation renal impairment is traditionally attributed to calcineurin inhibitors (CNI), a key component of immunosuppressive regimens for patients undergoing liver transplantation[1, 3, 11, 12]. However, several pieces of recent evidence have suggested that the cause of renal impairment may be complex[4–10]. Therefore, we performed the first renal histopathological exploration of liver transplant recipients who underwent renal biopsy in mainland China. All the patients were recruited from Ren Ji Hospital, Shanghai Jiao Tong University School of Medicine.

## Methods

This study was approved by the Institutional Review Board of Renji Hospital, School of Medicine, Shanghai Jiao Tong University. This study included 14 patients who received liver transplantation and then underwent renal biopsy at our center between July 2018 and February 2021. Renal biopsy was considered when there was proteinuria of > 1 g per day and/or significant renal impairment (> 50% persistent increase in serum creatinine from baseline level or estimated glomerular filtration rate (eGFR) < 60 mL/min/1.73 m^2^ on at least two occasions). All patients were older than 18 years and had stable liver allograft function with normal coagulation times and blood platelet counts. Patient information was obtained from the hospital’s electronic database. Data included age, sex, transplant date, renal biopsy date, primary etiology of liver disease, presence of comorbidities, post-transplant medications, and laboratory parameters at the time of liver transplantation. Renal biopsies were collected, including the levels of serum creatinine, blood urea nitrogen (BUN), estimated glomerular filtration rate (eGFR), uric acid (UA), urinary albumin/creatinine ratio (UACR), 24-hour urinary protein, urinary ɑ1-microglobulin, urinary β2-microglobulin, serum albumin, alanine aminotransferase (ALT), aspartate aminotransferase (AST), total bilirubin, international normalized ratio (INR), activated partial thromboplastin time (aPTT), platelet count, glycosylated hemoglobin (HbA1c), serum complement 3 (C3), serum complement 4 (C4), antinuclear antibodies (ANA) titer, serum IgA, serum IgM, serum IgG, and rheumatoid factor (RF), as well as hemoglobin (Hb) pre-and post-renal biopsy. Each patient was followed until April 2022.

## Renal biopsy

A standard percutaneous kidney biopsy was performed under ultrasound guidance as an inpatient procedure. Biopsy specimens prepared for light microscopy (LM) were formalin fixed, paraffin embedded, and stained with Periodic Acid Schiff reagents. Glutaraldehyde-fixed tissue was processed for electron microscopy (EM). Routine immunofluorescence studies were also done using polyclonal antibodies to IgA, IgG, IgM, albumin, C3, C4, C1q, and lambda and kappa light chain by standard methods[6]. All of the slides were reviewed by two experienced pathologists blind to the clinical and laboratory data. All biopsies contained at least 15 glomeruli.

## Statistical analyses

Clinical and biochemical characteristics of patients are expressed as the mean ± standard deviation (SD) or median [minimum (min.)–maximum (max.)], as appropriate. Data were evaluated using t-tests or Mann-Whitney nonparametric tests, as applicable. Correlations between two variables were calculated by Pearson coefficient, and p-values ≤ 0.05 were regarded as significant.

## Results

Overall, 14 renal biopsies were retrieved for review from 14 patients. The median age at the time of renal biopsy was 55 (range: 31–75) years, and renal biopsy was performed at a median interval after liver transplantation of 35.7 (range: 2.80–134.73) months. Detailed patient characteristics at renal biopsy are reported in Tables 1 and 2. At the time of renal biopsy, 12 patients were receiving a CNI-based immunosuppression protocol. One patient was on a combination of an mTOR inhibitor and a mycophenolate mofetil (MMF) immunosuppression regimen. One patient received an mTOR inhibitor only (CNI treatment was discontinued six months before the renal biopsy due to hepatocellular carcinoma relapse) (Table 1). Detailed information on risk factors of renal disease, immunosuppression, and major histological findings of renal biopsies is presented in Table 2. The predominant glomerular histomorphological changes included focal segmental glomerular sclerosis (FSGS) (n = 4), diabetic glomerulopathy (n = 4), and membranoproliferative glomerulonephritis (n = 4). Of note, immune complex nephritis was present in six patients, of whom four had membranoproliferative glomerulonephritis (n = 4) and two had IgA nephropathy (n = 2). Only two of these six patients had abnormal serum immunological indicators. Immune complex nephritis co-existed with diabetic glomerulopathy and hypertensive nephrosclerosis. Glomerular microangiopathy was present in one patient, which was considered lenvatinib-related nephropathy. Thrombotic microangiopathy(TMA ) was present in one patient and was considered a result of malignant hypertension.

**Table 1:** Clinical characteristics of patients with renal biopsy after liver transplantation

Parameter	Patients(n = 14)
Demography	
Age, years; median(min-max)	55(31–75)
Gender distribution, male/female	13/1
Interval between liver transplantation and kidney biopsy, years; median(min-max)	35.7(2.80-134.73)
Etiology of liver disease prior liver transplantation	
HBV, n(%)	12(85.7%)
Schistosomiasis, n(%)	1(7.1%)
Primary sclerosing cholangitis, n(%)	1(7.1%)
Comorbidity	
hypertension, n(%)	10(71.4%)
diabetes, n(%)	5(35.7%)
hepatocellular carcinoma, n(%)	8(57.1%)
Immunosuppression protocol at the time of renal biopsy	
CNI/MMF	5(35.7%)
CNI/mTOR inhibitor	4(28.6%)
CNI/MMF/prednisone	1(7.1%)
CNI	2(14.3%)
mTOR inhibitor/MMF	1(7.1%)
mTOR inhibitor	1(7.1%)
Biochemistry at the time of liver transplantation	
Scr, umol/L; median(min-max)	75.0(41.6, 129.3)
eGFR-EPI, mL/min/1.73m^2^;median(min-max)	103.9(55.2, 177.8)
Biochemistry at the time of renal biopsy	
Scr, umol/L; median(min-max)	118.5(86.0, 232.0)
BUN, mmol/L; median(min-max)	8.10(4.80, 14.40)
eGFR-EPI, mL/min/1.73m^2^;median(min-max)	48.5(26.0, 81.0)
Proteinuria(g/24 h), median(min-max)	3128.7(126.4,11183.7)
UACR(mg/g),median(min-max)	2318.1(25.60,4868.50)
Urine ɑ1-microglobulin(mg/L),median(min-max)	28.9(0.00, 51.70)
Urine β2-microglobulin(mg/L),median(min-max)	1.80(0.00, 15.30)
Serum albumin(g/L),median(min-max)	32.6(26.6, 46.1)
Uric acid(umol/L),median(min-max)	417.5(296.0, 667.2)
ALT(U/L),median(min-max)	13.0(3.0, 28.0)
AST(U/L),median(min-max)	17.0(10.0, 37.0)
TB(umol/L),median(min-max)	7.95(2.70, 45.80)
INR,median(min-max)	0.91(0.75, 1.08)
aPTT(s),median(min-max)	28.0(11.0, 30.7)
PLT(10^9/L),median(min-max)	132.0(79.0, 245.0)
HBA1c(%),median(min-max)	5.2(4.5, 6.8)
Abnormal Immunological indicators	
C3/C4 decreased	2(14.3%)
IgA/IgM/IgG increased	2(14.3%)
ANA positive	2(14.3%)
RF positive	1(7.1%)

HBV, hepatitis B virus; HCC, hepatocellular carcinoma; CNI, calcineurin inhibitor; MMF, mycophenolate mofetil; mTOR, mammalian target of rapamycin; Scr, serum creatinine; BUN, blood urea nitrogen; eGFR, estimated glomerular filtration rate; UACR, urine albumin/creatinine rate; ALT, alanine aminotransferase; AST, aspartate aminotransferase; TB, total bilirubin; INR, international normalized ratio; aPTT, activated partial thromboplastin time; PLT, platelet count; C3, serum complement 3; C4, serum complement 4; ANA, antinuclear antibodies; RF, rheumatoid factor; HBA1C, glycosylated hemoglobin

**Table 2:** Correlation of risk factors for kidney disease, immunosuppression, and histological alterations in patients with kidney biopsy after liver transplantation

Patient	Gender	Age (years)	Interval between liver transplantation and kidney biopsy (years)	Primary etiology of liver disease	HTN	DM	HCC	Scr (umol/L)	BUN (mmol/L)	eGFR (ml/min/1.73m^2^)	Proteinuria (g/24 h)	UACR (mg/g)	Urine ɑ1-microglobulin (mg/L)	Urine β2-microglobulin (mg/L)	Serum albumin (g/L)	UA (umol/L)	HBA1C (%)
1	female	55	10.67	HBV	N	N	N	149	9.3	32	620.1	897.5	< 5.26	0.18	39	667.2	5.2
2	male	31	0.34	HBV	N	N	N	103	6	73	3242.4	1568.8	15.20	< 0.17	26.6	475	5.1
3	male	59	1.16	HBV	Y	Y	Y	205	12	29	4480.9	4868.5	45.70	0.71	29	326	4.5
4	male	55	0.87	HBV	Y	N	N	112	6.5	59	245.0	25.6	25.10	3.02	46.1	427	4.9
5	male	55	10.24	HBV	N	Y	N	142	5.6	45	5824.8	2528.1	34.80	13.10	29.7	379	6.4
6	Male	54	0.67	HBV	Y	Y	Y	164	14.4	37	165.1	29.1	40.30	0.40	40.2	441	4.9
7	Male	62	5.17	HBV	Y	N	Y	95	7	49	3868.8	2649.6	30.10	2.23	27.4	296	6.2
8	Male	53	3.79	HBV	Y	N	Y	140	6.3	46	4500	1375.1	19.00	1.96	33.7	525	5.2
9	Male	52	2.64	HBV	Y	Y	Y	232	13.1	26	1065.5	2391.5	42.60	2.48	30.8	338	5.3
10	Male	72	0.23	Schistosomiasis	Y	N	Y	86	4.8	81	2761.2	2244.7	34.2	15.3	40.7	314	6.0
11	Male	57	134.73	PSC	N	N	N	125.00	9.70	55.00	126.4	106.0	20.90	0.28	36.50	465.00	4.80
12	Male	63	86.10	HBV	Y	N	Y	106.00	9.20	64.00	11183.7	4013.5	27.70	5.39	29.00	407.00	6.80
13	Male	75	39.27	HBV	Y	N	Y	99.00	9.90	48.00	3015.00	2656.7	18.20	0.00	31.00	408.00	4.90
14	Male	53	20.73	HBV	Y	Y	N	97.00	6.30	70.00	6360.90	2998.0	51.70	1.63	34.70	444.00	5.40

HTN, hypertension; DM, diabetes mellitus; Scr, serum creatinine; eGFR, estimated glomerular filtration rate; BUN, blood urea nitrogen; UACR, urine albumin/creatinine rate; HBV, hepatitis B virus; HCC, hepatocellular carcinoma; PSC, primary sclerosing cholangitis; Y, yes; N, no

**Table 2:** **(**Continued)

Patient	CNI/ mTOR inhibitor	Respective trough level	ALT (U/L)	AST (U/L)	TB (umol/L)	INR	aPTT (s)	PLT (10*9/L)	Main histological findings	Abnormal Immunological indicators
1	CSA	C0 118.9 mg/ml C2 247.2 mg/ml	16	28	45.8	1.03	30.7	85	IgAN	C3 0.56 g/L(L), C4 0.09 g/L(L), IgA 5.44 g/L(H),IgG 22.30 g/L(H), ANA 1:80
2	TAC	3.00ng/ml	12	15	7.9	0.89	28.0	132	membranoproliferative glomerulonephritis	N
3	TAC/RIPA	TAC 1.40ng/ml RIPA 5.40ng/ml	12	13	2.7	0.91	28.6	113	membranoproliferative glomerulonephritis, renal arteriolar sclerosis, DKD	N
4	CSA	C0/C2: 79.70/606.40 mg/ml	15	20	16.7	0.87	27.5	244	glomerular sclerosis, renal arteriolar sclerosis	N
5	TAC	< 1.00ng/ml	8	10	18.3	0.97	11.00	79	FSGS, DKD	N
6	TAC/RIPA	5.5/9.6ng/ml	3	13	5.1	0.98	26.6	83	TMA	N
7	RIPA	8.2ng/ml	18	19	8	0.85	29.6	174	FSGS, renal arteriolar sclerosis	N
8	TAC	3.1ng/ml	21	20	4.2	0.95	27.6	177	Membranoproliferative glomerulonephritis	C3 0.85 g/L(L), RA 25.60 IU/mL(H)
9	RIPA	4.4ng/ml	11	15	3.4	0.75	28.7	182	DKD	ANA 1:80
10	TAC/RIPA	TAC 6.40ng/ml RIPA NA	24	37	14.7	0.94	29.3	239	FSGS, renal arteriolar sclerosis	N
11	CSA	C0 9.50 mg/ml C2 355.60 mg/ml	28	33	18.6	1.08	27.3	187	FSGS, severe interstitial fibrosis and tubular atrophy	IgA 18.7 g/L(H); IgM 91.4 g/L(H)
12	TAC	3.60ng/ml	8	14	7.6	0.84	26.4	245	glomerular microangiopathy, DKD, renal arteriolar sclerosis	N
13	TAC/RIPA	NA	14	35	6.8	0.95	30.0	89	membranoproliferative glomerulonephriti, renal arteriolar sclerosis	N
14	TAC	NA	12	14	12.9	0.88	22.1	98	IgAN	N

CNI, calcineurin inhibitor; mTOR, mammalian target of rapamycin; MMF, mycophenolate mofetil; CSA, cyclosporine A; ALT, alanine aminotransferase; AST, aspartate aminotransferase; TB, total bilirubin; INR, international normalized ratio; aPTT, activated partial thromboplastin time; PLT, platelet count; IgAN, IgA nephropathy; DKD, diabetes kidney disease; FSGS, focal segmental glomerular sclerosis; TMA, thrombotic microangiopathy; ANA, antinuclear antibodies; RF, rheumatoid factor; NA, not available

## Assessment of histological changes in kidney disease

The histological findings are summarized in Table 3. There was a mean of 28 ± 13 glomeruli per biopsy specimen. All biopsies demonstrated glomerular abnormalities. Most (57.1%) of the biopsies demonstrated severe glomerulosclerosis involving more than 40% of glomeruli. Mesangial matrix expansion was also prevalent and detected in 92.9% of the biopsies. Specific glomerular lesions were found, including ten (71.4%) focal segmental glomerulosclerosis, three (21.4%) membranoproliferative glomerulonephritis, two (14.3%) crescents, and one (7.1%) glomerular microangiopathy. Thirteen (92.9%) biopsy specimens had arterionephrosclerosis.

**Table 3:** Histopathologic findings on kidney biopsy after liver transplantation

Parameter	Patients(n = 14)
Glomerular abnormalities, n(%)	14(100%)
Glomerulosclerosis	
Mild(< 20%), n(%)	3(21.4%)
Moderate(20-40%), n(%)	3(21.4%)
Severe(> 40%), n(%)	8(57.1%)
Increased mesangial matrix, n(%)	13(92.9%)
Specific glomerular lesions	
Focal segmental glomurulosclerosis, n(%)	10(71.4%)
Membranoproliferative glomerulonephritis, n(%)	3(21.4%)
Glomerular microangiopathy	1(7.1%)
Cresents, n(%)	2(14.3%)
<50%, n(%)	1(7.1%)
≥50%, n(%)	1(7.1%)
Arterial abnormalities, n(%)	13(92.9%)
Tubulointerstitial abnormalities	14(100%)
Mild(< 25%), n(%)	2(14.3%)
Moderate(25-50%), n(%)	3(21.4%)
Moderate to Severe(50–75%)	3(21.4%)
Severe(> 75%), n(%)	6(42.9%)
Interstitial fibrosis, n(%)	13(92.9%)
Immunofluorescence abnormalities, of glomuruli, n(%)	10(92.9%)
IgA positive, n(%)	9(64.3%)
IgM positive, n(%)	6(42.0%)
IgG positive, n(%)	1(7.1%)
C3 positive, n(%)	8(57.1%)
C1q positive, n(%)	3(21.4%)

All biopsy specimens had tubulointerstitial abnormalities that were mild in two (14.3%), moderate in three (21.4%), moderate to severe in three (21.4%), and severe in six (42.9%) biopsies. Severe interstitial injury was present in six patients, five of whom were receiving a CNI-based immunosuppression protocol, while the other one was receiving mTOR inhibitor only (CNI treatment had been discontinued six months before renal biopsy due to hepatocellular carcinoma relapse). Of note, for patients receiving a CNI-based immunosuppression protocol, mild or moderate tubular atrophy and interstitial fibrosis were also present in four (33.3%) patients. There was a significant correlation between the severity of tubular atrophy and interstitial fibrosis and urine ɑ1-microglobulin (r = 0.601, p = 0.023).

Immunofluorescence was positive in 92.9% of the biopsies, and included nine (64.3%) that were IgA positive, six (42.0%) that were IgM positive, one (7.1%) that was IgG positive, eight (57.1%) that were C3 positive, and three (21.4%) that were C1q positive.

## Assessment of complications after renal biopsy

Renal biopsy was a safe procedure in our cohort as no major complications were observed. A mild decrease in Hb without symptoms was documented [pre- vs. post- renal biopsy 105.5 (84.0–162.0) g/L vs. 103.5 (82.0–157.0) g/L, p = 0.018] by routine tests three days after biopsy.

## Therapeutic consequences of kidney histology and clinical course

The mean follow-up time after kidney biopsy was 11.8 months (range: 1.2–29.8). The median serum creatinine at the last follow-up visit was 121.5 umol/L (range: 72.0–652.0), median eGFR was 54.8 (range: 8.0–94.0) mL/min/1.73 m2, and median serum albumin was 36.9 g/L (range: 25.9–46.9). Three patients progressed to end-stage renal disease(ESRD)(Table 4). For those patients who progressed to ESRD, serum creatinine at the time of renal biopsy was 140–205 umol/L, presenting 50–60% glomerulosclerosis.

**Table 4:** Clinical outcomes of the patient population

Parameter	Patients(n = 14)
Median follow-up, months; median(min-max)	11.8(1.2–29.8)
Mortality, n(%)	2(14.3%)
ESRD	3(21.4%)
Biochemistry at last follow-up	
Scr, umol/L; median(min-max)	121.5(72.0, 652.0)
eGFR-EPI, mL/min/1.73m^2^;median(min-max)	54.8(8.0, 94.0)
Alb, g/L; median(min-max)	36.9(25.9, 46.9)

ESRD, end-stage renal disease; Scr, serum creatinine; eGFR, estimated glomerular filtration rate; Alb, albumin

## Discussion

Correctly diagnosing and optimally treating renal impairment after liver transplantation remains challenging worldwide. Although CNI-induced nephrotoxicity is a generally acknowledged common cause of renal impairment post liver transplantation, it is, obviously, not the only potential etiology. Moreover, there was no significant renal function improvement after withdrawal of CNI[3, 13, 14]. The underlying etiology is multifactorial. Amongst many risk factors the most important ones include immuosuppression-related nephrotoxicity, renal function impairment before Liver transplantation, hepatitis virus infection, hypertension, diabetes, metabolic syndrome and obesity. Several data about renal biopsy after liver transplantation have been reported in recent years. Despite relatively small sample sizes (from 4 to 81 patients) and retrospective study designs, most of them have suggested that the etiology of CKD after liver transplantation is broad and complex and may only be assessed accurately by renal biopsy[4–10]. However, the importance of renal biopsy remains underestimated by clinicians, especially in mainland China. To the best of our knowledge, the current study is the first to analyze the histopathology in CKD patients after liver transplantation in mainland China.

CNIs remain important but are not the only cause of kidney disease in the liver transplant population, and the search for less toxic immunosuppressive agents remains an ultimate goal. In our cohort, severe interstitial injury was present in six (42.9%) patients, five of whom were receiving a CNI-based immunosuppression protocol. They were routinely monitored for serum drug levels and modification of immunosuppressive therapy. The other one patient was receiving mTOR inhibitor only (CNI treatment was discontinued six months before renal biopsy due to hepatocellular carcinoma relapse).

Diabetes nephropathy was present in four (28.6%) patients, all of whom had a relatively normal range of HBA1C (4.5–6.4%). This finding was consistent with a previous report stating that diabetes nephropathy is common in CKD after liver transplantation, even in patients without a diabetes history[6]. Renal arteriolar sclerosis was presented in 13 (92.9%) patients. This finding might lead to more aggressive treatment of diabetes and hypertension.

Unexpectedly, we diagnosed lenvatinib-related nephropathy with a histopathologic diagnosis of glomerular microangiopathy, which was first reported in this population. After withdrawing lenvatinib, partial remission was achieved in one month. TMA was present in one patient, which was considered a result of malignant hypertension. Renal function partially recovered after good control of hypertension was achieved. In these cases, the results of the renal biopsies enabled specific nephrology treatment and helped avoid unnecessary modification of immunosuppression.

Immune complex nephritis was evident in six biopsies, namely membranoproliferative glomerulonephritis (n = 4) and IgA nephropathy (n = 2). Serum C3 decreased in two of them, while the others had normal immunological indicators. Notably, immune complex nephritis co-existed with diabetic glomerulopathy and hypertensive nephrosclerosis, resulting significantly in a correct diagnosis without renal biopsy. However, the cause of immune complex nephritis (primary or secondary to infection, tumor, HBV, drugs, or even a result of transplantation immune response) in this population remains ambiguous. In addition, more accurate diagnostic methods need to be explored.

In our cohort, hepatocellular carcinoma was presented in eight (57.1%) patients using various antineoplastic treatments, including tyrosine kinase inhibitor, platinum, and radiotherapy. The nephrotoxicity of antineoplastic agents has been reported in recent years[15–18], but the histopathological evidence has been limited, especially in patients after liver transplantation.

After a median follow-up of 11.8 months post-renal biopsy, three patients had developed ESRD, and two had died. Such data were comparable to those from a previous series [4, 6, 7]. Notably, for those patients who progressed to ESRD, serum creatinine at the time of renal biopsy was 140–205 umol/L, presenting 50–60% glomerulosclerosis. Therefore, a delayed renal biopsy might barely be of benefit for prognosis. We encourage kidney biopsies to be performed more frequently and early in patients with renal impairment after liver transplantation.

Our study has several limitations. It was a single-center, retrospective-design case series, which limits the generalizability of the results. Furthermore, the sample size was obviously limited, leading to relatively low statistical power, which may have rendered it insufficiently powered to compare outcomes and adjusted confounders. Besides, there was a great possibility of bias in this current study. We cannot give a true representation of the percentage of live transplantation recipients referred for renal review as not all the patients accepted renal biopsy. On the other hand, a number of surgeons might have insufficient awareness of various possibility of renal impairment in liver transplantation patients during their follow-up, resulting in late referral to nephrologists. Therefore, the results need to be interpreted with caution. Another important drawback is the lack of practice-changing knowledge. Meanwhile, published studies evaluating renal pathology after liver transplantation are sparse, and all of them had small sample sizes. Moreover, data about renal pathology after liver transplantation has not reported in mailand China in the last few dacades. Considering the above shortcomings, we will conduct a prospective cohort study with a relatively large sample size to understand the pathology of kidney diseases and to explore the risk factors of renal function progression in post-liver transplantation patients with renal impairment(NCT 05326399).

## Conclusion

The etiology of CKD might be more complex after liver transplantation than originally thought and should not simply be diagnosed as CNI-related nephropathy. Renal biopsy plays a potentially important role in the diagnosis of CKD after liver transplantation, which might not be fully substituted by urine or blood tests. Renal biopsies might provide a potential method to help avoid unnecessary changes to immunosuppressants and inadequate treatment of primary diseases.

## Data Availability

All data generated or analysed during this study are included in this article. Further enquiries can be directed to the corresponding author.
